# Alteraciones Conductuales en Adolescentes y Adultos Jóvenes con Trastorno del Espectro Autista en Unidad de Hospitalización: Análisis de las Autolesiones

**DOI:** 10.31083/RN45344

**Published:** 2025-07-28

**Authors:** Berta Massaguer-Bardají, Antoni Grau-Touriño, Antonia Maria Gómez-Hinojosa

**Affiliations:** ^1^CSMIJ de Granollers, Hospital Sant Joan de Déu, 08950 Esplugues de Llobregat, España; ^2^Facultat de Psicologia, Ciències de l’Educació i l’Esport Blanquerna, 08022 Barcelona, España; ^3^ITA Especialistas en Salud Mental, 28007 Madrid, España

**Keywords:** autolesión, Trastorno del Espectro Autista (TEA), ansiedad, depresión, defensividad sensorial, self-harm, Autism Spectrum Disorder (ASD), anxiety, depression, sensory defensiveness

## Abstract

**Antecedentes:**

Las personas con diagnóstico de Trastorno del Espectro Autista (TEA), pueden presentar alteraciones en la conducta, dificultades para el cambio, intereses restringidos y/o alteraciones sensoriales. Entre sus comportamientos característicos se encuentran conductas autolesivas que tienden a ser compulsivas, no planificadas, rítmicas y repetitivas. Ante ello, se planteó como objetivo establecer la correlación existente entre las conductas autolesivas en adolescentes hospitalizados con TEA, la depresión y la ansiedad.

**Método:**

La muestra incluyó 50 pacientes con TEA, entre los 14 y los 27 años. A estos pacientes se les aplicó la Escala de Observación para el Diagnóstico de Autismo (ADOS-2), la Entrevista para el diagnóstico de Autismo – Revisada (ADI-R), Cuestionario de ansiedad estado-rasgo, Inventario de Depresión de Beck (BDI), cuestionario Adolescent/Adult Sensory Profile (AASP) e “Inventory of Statements About Self-injury (ISAS)”.

**Resultados:**

Los resultados revelaron correlaciones significativas y positivas entre el nivel de autolesión y sus dimensiones: autorregulación (ρ = 0,861), búsqueda de sensaciones y fortaleza (ρ = 0,767), evitación del suicidio (ρ = 0,732), venganza (ρ = 0,643), autodominio (ρ = 0,700), manifestación de angustia (ρ = 0,898) y embotamiento (ρ = 0,702).

**Conclusiones:**

Se evidencia una relación positiva entre los niveles de autolesión y la defensividad sensorial, específicamente en el perfil evitador de emociones.

## Abstract (en)

**Background:**

Individuals with Autism Spectrum Disorder (ASD), may present with behavioral disturbances, difficulties dealing with change, restricted interests and/or sensory disturbances. Among their characteristic behaviors are self-injurious behaviors that tend to be compulsive, unplanned, rhythmic and repetitive. The aim of this study was to investigate the relationship between self-injurious behaviors in hospitalized adolescents with ASD, depression and anxiety.

**Methods:**

The sample included 50 patients with ASD, aged between 14 and 27 years. These patients were assessed using the Autism Diagnostic Observation Scale (ADOS-2), the Autism Diagnostic Interview-Revised (ADI-R), the Trait-State Anxiety Questionnaire, the Beck Depression Inventory (BDI), the Adolescent/Adult Sensory Profile (AASP) and the Inventory of Statements About Self-injury (ISAS).

**Results:**

The results revealed significant and positive correlations between the level of self-injury and its dimensions: self-regulation (ρ = 0.861), sensation seeking and strength (ρ = 0.767), suicide avoidance (ρ = 0.732), revenge (ρ = 0.643), self-control (ρ = 0.700), manifestation of distress (ρ = 0.828) and blunting (ρ = 0.702).

**Conclusions:**

There is evidence of a positive relationship between levels of self-harm and sensory defensiveness, specifically in the emotion avoidance profile.

## 1. Introducción

El Trastorno del Espectro Autista (TEA) es un trastorno del neurodesarrollo que se manifiesta desde la infancia temprana y afecta de forma significativa el desarrollo de la comunicación, la interacción social recíproca y la conducta. Se caracteriza por alteraciones cualitativas en las habilidades sociales y comunicativas, así como por la presencia de comportamientos repetitivos e intereses restringidos y persistentes [1, 2].

La evolución de este trastorno es crónica o mantenida a lo largo del desarrollo y se manifiesta en diversos niveles de afectación, adaptación funcional, competencias lingüísticas y cognitivas. Entre los diferentes niveles de gravedad, según lo expresado en el manual diagnóstico y estadístico de los trastornos mentales (DSM-5) [3], se encuentran:

(a) Dentro de la normalidad: cuando no presenta interferencia en el comportamiento, ni en la comunicación, aunque la misma es peculiar o aislada.

(b) Con síntomas subclínicos: presentan pequeñas alteraciones, aunque no significativas, tanto en su comunicación social como en su comportamiento.

(c) De grado 1 “necesita ayuda”: presenta algunas alteraciones significativas en el ámbito de la comunicación, pero sin apoyo *in situ*, así como pequeñas interferencias de su conducta.

(d) De grado 2 “necesita ayuda notable”: tiene un marcado déficit en respuestas comunicativas e interferencias frecuentes de la conducta, caracterizadas por inflexibilidad y dificultades de aceptación.

(e) De grado 3 “necesita ayuda muy notable”: presenta una mínima comunicación social y marcada interferencia en las acciones de la vida cotidiana además de dificultades de cambio en el foco de atención y la inflexibilidad.

Como se ha mencionado anteriormente, las personas con TEA se caracterizan por presentar alteraciones o déficits de interacción y comunicación social. Frente a esto, destacan las dificultades en la reciprocidad socioemocional, la comunicación verbal o no verbal y el desarrollo y sostenimiento de relaciones sociales. De igual manera, presentan alteraciones en sus intereses y en la conducta, las cuales, se evidencian por medio de comportamientos repetitivos, dificultades para el cambio, intereses restringidos y/o alteraciones sensoriales [3].

En correspondencia con esto, Kim [4], precisa que, entre las manifestaciones clínicas que presentan los individuos con este trastorno, se encuentran patrones de conducta restringidos y repetitivos, que se caracterizan por: estereotipias, comportamientos impulsivos y autolesivos; repeticiones continuas de una misma interrogante sin que importe la respuesta que hayan obtenido a la misma; y, participación en juegos de carácter repetitivos, los cuales, en ocasiones, podrían ser considerados aspectos de preocupación en el entorno familiar y escolar. Así mismo, presentan dificultades en la consolidación de interacciones sociales, debido principalmente a la escasa demostración de afecto al socializar con otros. Esta característica reitera el escaso interés social, la carencia de amigos o escogencia selectiva de los mismos, así como, la preferencia por jugar en solitario. Por otra parte, se caracterizan por un marcado deterioro en los procesos comunicativos, principalmente, reflejados a través de balbuceos (durante la primera infancia), los cuales, no logran ser indemnizados con gestualizaciones, así como, ecolalia inmediata o retardada [5].

Uno de los comportamientos de esta población, que ha llamado la atención en los últimos tiempos, ha sido las conductas autolesivas, que conllevan un importante deterioro de la salud del individuo, así como, de su calidad de vida. También implica una limitación de relevancia para el desarrollo de las capacidades y habilidades, lo cual, lleva implícito el desarrollo integral del individuo [5].

Al hablar de autolesiones, se hace referencia a un concepto polisémico, cuya definición varía según el enfoque clínico, social o cultural desde el que se aborde [6]. En términos generales, se entiende como cualquier acto deliberado que un individuo realiza contra sí mismo con la intención de infligirse daño físico, sin que exista una intención suicida explícita y sin que necesariamente tenga un desenlace fatal [7, 8]. Este comportamiento, que incluye acciones como cortes, quemaduras o golpes autoinfligidos, suele ser interpretado como una estrategia de regulación emocional frente a estados intensos de angustia, disociación o vacío afectivo [9].

Aunque en muchas ocasiones no tiene consecuencias letales, la autolesión es sancionada y estigmatizada desde el punto de vista cultural, y plantea desafíos tanto para la intervención clínica como para la comprensión social del sufrimiento psíquico [10]. Además, su manifestación está frecuentemente asociada a trastornos del neurodesarrollo, como el TEA, y a otras condiciones psicopatológicas, donde puede adquirir una función comunicativa o adaptativa en ausencia de habilidades emocionales más elaboradas [11, 12].

Las personas que tienen un diagnóstico de TEA suelen tener inclinaciones a realizar autolesiones no suicidas, es decir, acciones que conllevan daño directo y deliberado contra el organismo propio, aunque, en ausencia de la intencionalidad suicida [13].

Estos comportamientos autolesivos, en quienes han sido diagnosticados con autismo, tienden a ser compulsivos, no planificados, rítmicos y repetitivos. Un ejemplo de estas conductas es el golpearse de manera repetida en un mismo punto durante un tiempo determinado. La intensidad o la fuerza que los individuos con autismo normalmente ejercen al realizar la autolesión, depende del nivel de estrés que presente en el momento, por lo cual, puede señalarse que existe una relación directa entre el estado de alerta y el nivel de ansiedad, con la calidad de la conducta autolesiva. Este tipo de conducta suele presentarse en individuos con un grado de autismo grave [13].

Algunos estudios se han realizado sobre los planteamientos anteriores. Entre estos se encuentra el estudio realizado por Tudela Torras y Abad Más [13], sobre la reducción de conductas autolesivas y autoestimulatorias disfuncionales en individuos con TEA, para lo cual, se llevó a cabo una revisión bibliográfica sobre las diversas líneas de atención a las conductas autolesivas que se han implementado hasta el momento y se expone una metodología fundamentada en la terapia ocupacional que se aplica con el fin de observar si existe disminución de estas conductas. Los datos del estudio presentan una recopilación de información sobre el efecto de la terapia ocupacional sobre las conductas autolesiva y autoestimulatorias. Como conclusión, se destaca el modelo de integración sensorial, el cual, complementa el tratamiento farmacológico y la terapia cognitivo conductual, debido a que, toma en cuenta como fundamento las necesidades sensoriales del individuo y lo capacita para lograr una autorregulación funcional.

Otro de los estudios ha sido el desarrollado por Castro Silva *et al*. [14] a través del cual se evaluó la adaptación y las propiedades psicométricas de la población estudiantil de habla hispana. La escala evalúa las diversas motivaciones que los individuos refieren para realizar autolesiones no suicidas. Se aplicó a una muestra de 435 estudiantes universitarios de ambos sexos que ya poseían historia previa de autolesiones no suicidas. A través de un análisis factorial se confirmaron y detectaron siete [7] factores interpretables, como son: la autorregulación, la venganza, la búsqueda de sensaciones o fortalezas, la evitación del suicidio, la angustia, la autodeterminación y el embotamiento. La confiabilidad de la escala fue aceptable, con un alfa de Chrome de 0,089 para la escala total y entre 0,072 y 0,082 para cada factor. Se presentó de igual modo la validez convergente por medio de correlaciones positivas entre la escala y mediciones de depresión, ansiedad e impulsividad.

Se concluye que el instrumento adaptado al español presenta propiedades psicométricas aceptables para la medición de autolesiones no suicida en la población universitaria mexicana no TEA. Resulta relevante mencionar esta escala porque identifica múltiples factores motivacionales de la autolesión, muchos de los cuales también pueden estar presentes en personas con TEA. Aunque fue validada en población universitaria sin TEA, sus propiedades psicométricas y su enfoque multidimensional ofrecen un referente útil para comprender y analizar las funciones de la autolesión, y subrayan la importancia de adaptar y validar instrumentos específicos para poblaciones neurodivergentes.

Otro de los estudios encontrados es un análisis psicométrico de funcionamiento en una muestra de drogodependientes. Para ello, se aplicó el instrumento Cuestionario de ansiedad estado-rasgo (state-trait anxiety inventory (STAI)). Esta investigación fue desarrollada por del Río Olvera *et al*. [15] con el propósito de evaluar las propiedades psicométricas del cuestionario y comparar sus resultados con una población no clínica. Para la elección de la muestra se aplicó un muestreo por conglomerados en 28 centros de tratamiento para las adicciones, distribuidos en 27 provincias españolas. La muestra final estuvo constituida por 1054 individuos consumidores de sustancias y 211 no consumidores. El análisis de la fiabilidad evidenció una adecuada validez interna, por su parte, el análisis de los ítems agrega la necesidad de revisar dos de ellos cuando se emplea en población con historial de consumo de sustancias adictivas.

Los resultados evidencian que, los individuos drogodependientes obtuvieron una puntuación media superior a las no dependientes, así como, que las mujeres obtuvieron una puntuación media superior en relación con los hombres y que los individuos que habían consumido sustancias depresoras obtuvieron una puntuación superior a quienes habían consumido sustancias estimulantes, concluyendo que las diferencias fueron estadísticamente significativas. Por tanto, la escala STAI es relevante en este estudio porque permite evaluar la ansiedad, un factor clave vinculado a las autolesiones en personas con TEA. Su validez interna y sensibilidad ante diferencias clínicas refuerzan su utilidad como referente para futuras adaptaciones en poblaciones neurodivergentes.

En función de todo lo anterior se determinó como pregunta de investigación ¿cuál es la relación existente entre el nivel de autolesiones de los adolescentes hospitalizados con autismo y los estados de depresión y ansiedad? Frente a esto, se propuso como objetivo general establecer la correlación existente entre el nivel de autolesiones en adolescentes hospitalizados que presentan un TEA, la depresión y la ansiedad. Para culminar se propusieron las siguientes hipótesis.

Hipótesis 1: “Existe una correlación positiva entre los grados de ansiedad y depresión y el nivel o grado de afectación del autismo”.

Hipótesis 2: “Existe una correlación positiva entre los grados de ansiedad y depresión y el nivel de autolesión”.

Hipótesis 3: “Existe una correlación positiva entre los grados de autolesión y la defensividad sensorial”.

## 2. Método

### 2.1 Muestra

**Fig. 1.**
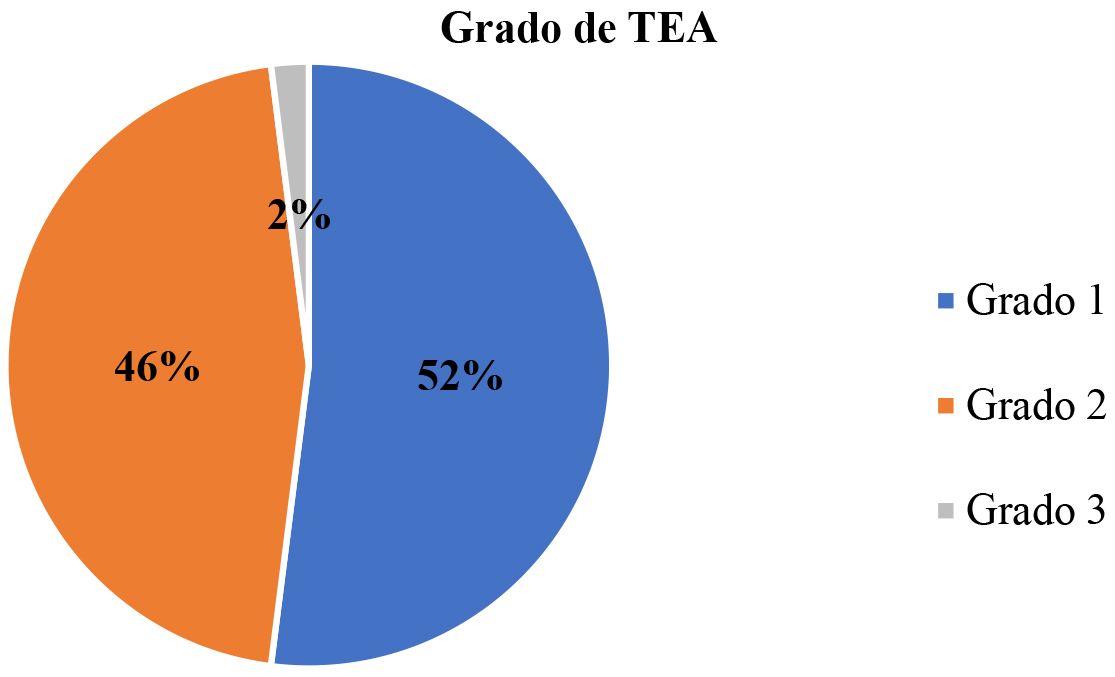
**Distribución por grado de Trastorno del Espectro Autista (TEA)**.

La muestra estuvo integrada por un total de 50 pacientes con TEA (28 de género masculino correspondiente a 56% y 22 de género femenino equivalente al 44%), con edades comprendidas entre los 14 y los 27 años, nacidos entre 1996 y 2009. Del total de participantes, 46% viven con su familia de origen completa, 20% con madre y hermanos, 10% en centro de acogida, 12% sólo con la madre y 12% otros convivientes. El 46% (23 participantes) ha recibido escolarización con soporte. En relación con el grado de autismo otorgado en el diagnóstico se ha observado que 52% (26 participantes) tiene grado 1; 46% (23 participantes) tienen grado 2 y sólo 2% (1 participante) fue diagnosticado con grado 3, como se describe en la siguiente figura (Fig. 1).

El 66%, equivalente a 33 participantes, tiene hermanos, de los cuales, 72% ocupan el primer lugar en el orden de nacimiento, 26% ocupa el segundo lugar, 2% ocupa el tercer lugar. El 92% se encuentra ingresado en ITA Especialistas en Salud Mental. De ellos, el 86% ha tenido ingresos previos en el centro hospitalario (26% solo 1 vez, el mismo porcentaje 2 veces; 18% ha ingresado 3 veces; 8% ha ingresado 4 veces).

Por otra parte, el 66% realiza actividades extraescolares. De ellos, el 34% practican actividades deportivas, 24% artísticas y 8% culturales (Tabla 1). El 70% (35 participantes) tiene discapacidad. Para el 12% de ellos la discapacidad es de grado 36, para el 10% es de grado 33, para el 6% de grado 42. El mismo porcentaje para quienes tienen grado 65 y 67.

**Tabla 1.** **Resultados sobre actividades extraescolares**.

	Si		No	
	N	%	N	%
Actividades extraescolares	33	66%	17	34%
Actividades deportivas	17	34%		
Actividades artísticas	12	24%		
Actividades culturales	4	8%		

El 16% (8 participantes) a nivel legal, cuentan con una modificación de la capacidad de obrar y un 26% presentan de una enfermedad orgánica. De ellos un 6% sufren de epilepsia, un 4% de diabetes tipo II, la misma cantidad hipertiroidismo, un 2% Diabetes tipo I, 2% epilepsia e hipertiroidismo, 2% hipotiroidismo, 2% Síndrome de Crigler-Najjar y 2% Síndrome de Wilkie.

El 42% (21 participantes) provienen del hospital, un 34% (17 participantes) provienen de su domicilio, 12% de un centro terapéutico y 12% (6 participantes) procedentes de un centro gestionado por la “direcció general d’atenció a la infància i adolescència” (DGAIA).

**Fig. 2.**
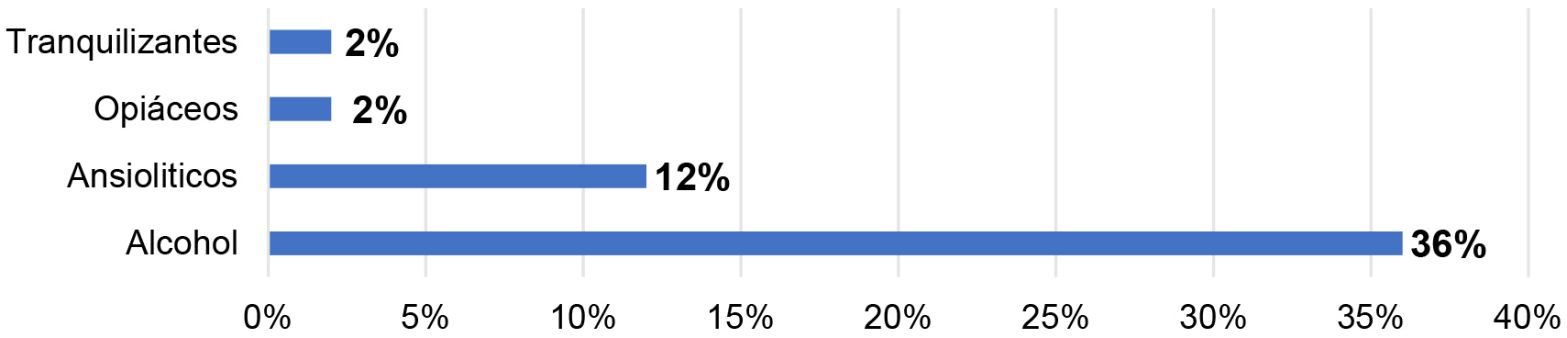
**Consumo de sustancias depresoras del sistema nervioso**.

Por otro lado, el 38% consumen sustancias de tipo legal y 26% de tipo ilegal. Sobre el consumo de sustancias depresoras del sistema nervioso, podemos apreciar que el 36% consumen alcohol, 12% ansiolíticos, 2% opiáceos y 2% tranquilizantes (Fig. 2).

Sobre las estimulantes del sistema nervioso: el 44% utiliza nicotina y un 2% anfetaminas. Asimismo, un 14% consume cannabinoides (sustancia psicodélica). En cuanto a la medicación un 46% utilizan antipsicóticos, 52% ansiolíticos, 38% antidepresivos, 26% estabilizadores de ánimo y psico-estimulantes.

Respecto a la muestra, se realizó un índice de correlación entre las variables de ansiedad, depresión y diagnóstico de autismo con sus dimensiones, medidas por la Escala de Observación para el Diagnóstico de Autismo – Segunda Edición ADOS-2 que se administró a los pacientes de la muestra.

Se analizaron las correlaciones existentes entre el nivel de autolesión y ansiedad, así como la autolesión y la depresión.

### 2.2 Instrumentos de Medición 

Para esta investigación se utilizó la prueba de observación clínica ADOS-2. Cuenta con 5 módulos, cada uno de los cuales está destinado a personas con diferentes edades cronológicas y niveles de lenguaje. Para efectos de este estudio se utilizó el 4to módulo, dispuesto para adultos y adolescentes de 16 años en adelante con lenguaje fluido. Cuenta con una fiabilidad de 0,88 (total global del algoritmo), 0,87 (Afectación social) y 0,64 (comportamientos restringidos y repetitivos) [15].

También se aplicó “la entrevista para el diagnóstico de autismo – revisada” (ADI-R) que consiste en una entrevista estándar semiestructurada, aplicada a padres y/o cuidadores de personas con posibilidad de autismo y se estructura partiendo de los criterios del DSM-5 y la clasificación internacional de enfermedades (CIE-10). Genera puntuaciones para algoritmos de los tres principales dominios del espectro autista: trastornos cualitativos de la conducta social recíproca, retrasos en el desarrollo del lenguaje y conductas estereotipadas e intereses restringidos. Está compuesta por 93 ítems y cuenta con una consistencia interna que varía, según el estudio, entre 0,63 a 0,89 y los coeficientes de correlación intraclase se encuentran por encima de 0,92 para todos los dominios y subdominios [16].

Así mismo, se aplicó el “Cuestionario de ansiedad estado-rasgo (STAI)” que consiste en un cuestionario autoaplicable conformado por 40 ítems. Este instrumento evalúa dos conceptos independientes de la ansiedad: la ansiedad como estado (condición emocional transitoria) y la ansiedad como rasgo (propensión ansiosa relativamente estable). Cuenta con una consistencia interna que oscila, tanto para la puntuación total como para cada una de las subescalas, entre 0,84 y 0,93.

Otro de los instrumentos aplicados fue el inventario de depresión de Beck (BDI). Es una escala autoaplicable que consta de 21 ítems, indicativos de síntomas como tristeza, lloro, pérdida de placer, sentimiento de fracaso y de culpa, pensamientos de deseo o deseo de suicidio, etc. Este instrumento evalúa la gravedad de sintomatología depresiva en adultos y adolescentes con una edad mínima de 13 años. Cuenta con una alta consistencia interna con un coeficiente alfa de alrededor superior a 0,85, tanto en muestras clínicas como no clínicas [17].

Se aplicó también el cuestionario “adolescent/adult sensory profile (AASP)” que permite estimar los rasgos de los distintos patrones de procesamiento sensorial y efectos en el comportamiento de la vida diaria. Este instrumento traducido cuenta con una consistencia con puntuaciones que se sitúan entre 0,69 y 0,73 [18, 19].

Finalmente, se implementó el “Iinventory of statements about self-injury (ISAS)” [20], versión en español de Castro Silva *et al*. [14] consistente en 39 ítems que componen 13 funciones, las cuales, se agrupan dos factores: interpersonal e intrapersonal. La prueba original tiene una consistencia interna de 0,84 para el apartado de comportamientos y de 0,88 para el apartado de funciones [20].

### 2.3 Procedimiento

Para la comprobación de las hipótesis, se aplicó el coeficiente correlación de Spearman (Rho de Spearman) mediante el uso del programa estadístico IBM *SPSS Statistics* (versión 26, IBM Corp., Armonk, NY, USA) con un nivel de significancia de 5%.

Primeramente, se realizó el análisis correlacional entre las variables ansiedad y depresión y el nivel de afectación o diagnóstico de autismo. A continuación, se realizó el análisis correlacional entre las variables ansiedad y depresión y el nivel de autolesión. Finalmente, se aplicó el análisis correlacional para determinar la relación entre las variables autolesión y la defensividad sensorial.

## 3. Resultados

En el al análisis descriptivo, las puntuaciones de ansiedad permitieron evidenciar una media de 46,58 (mínima de 16 y máxima de 80) pudiendo interpretarse que en promedio los pacientes adolescentes con TEA sufren de ansiedad mayor. En el análisis de depresión se observó una media de 18,52 (mínima de 3 y máxima de 37) interpretándose que, en promedio, los participantes sufren de depresión leve. Finalmente, en el análisis de autolesiones se observó una media de 9,6 (mínima 0,00 y máxima 38,00), detallando un bajo índice de autolesión (Tabla 1).

Entre las dimensiones de la autolesión se observa mayor puntuación en la autorregulación con una media de 2,74, seguido por la manifestación de angustia con media de 1,74, búsqueda de sensaciones/fortaleza con media de 1,62 y evitación del suicidio con media de 1,35.

Los resultados del análisis correlacional evidencian asociaciones positivas y significativas entre las variables que se mencionan a continuación: la depresión y la ansiedad ρ = 0,467; entre los resultados del diagnóstico de autismo y cada uno de sus componentes (Comunicación ρ = 0,513; Interacción Social Recíproca ρ = 0,840; Comunicación + Interacción Social Recíproca ρ = 0,900; imaginación y creatividad ρ = 0,413; Comportamientos estereotipados e intereses restringidos ρ = 0,585) (Tabla 2). No obstante, no se observó ninguna significación entre la ansiedad y la depresión con los niveles de autismo ni con el género. Solamente se pudo encontrar una asociación negativa entre la depresión y el componente de imaginación y creatividad dentro del Trastorno del Espectro Autista.

**Tabla 2.** **Índice de correlación de las variables ansiedad, depresión y diagnóstico de autismo con sus dimensiones**.

	Ansiedad (STAID)	Depresión (BDI)	Diagnóstico de Autismo	Comunicación	Interacción social recíproca	Comunicación + Interacción social recíproca	Imaginación + creatividad	Comportamientos estereotipados e intereses restringidos
Ansiedad (STAID)	1							
Depresión (BDI)	0,467**	1						
Diagnóstico de Autismo	–0,122	–0,082	1					
Comunicación	–0,219	–0,142	0,513**	1				
Interacción social recíproca	–0,014	0,019	0,840**	0,215	1			
Comunicación + Interacción social recíproca	–0,106	–0,049	0,900**	0,608*	0,903**	1		
Imaginación + creatividad	–0,237	–0,291	0,413**	0,003	0,225	0,180	1	
Comportamientos estereotipados e intereses restringidos	0,006	0,091	0,585**	0,286*	0,286*	0,309*	0,289	1

Nota. Autoría propia a partir de los datos obtenidos en el proceso de investigación. ** La correlación es significativa en el nivel 0,01 (bilateral). * La correlación es significativa en el nivel 0,05 (bilateral).

Por otra parte, se observaron también asociaciones significativas y positivas entre el nivel de autolesión y el de ansiedad ρ = 0,480, así como, autolesión y depresión ρ = 0,429. En las dimensiones de la autolesión entre el nivel de depresión y el de autorregulación ρ = 0,541, evitación de suicidio ρ = 0,508 y manifestación de angustia ρ = 0,392. También entre la ansiedad y la autorregulación ρ = 0,542. Del mismo modo se observaron correlaciones positivas entre la ansiedad y la búsqueda de sensaciones y fortalezas ρ = 0,340, la evitación de suicidio ρ = 0,360, la venganza ρ = 0,301, el autodominio ρ = 0,309; y la manifestación de angustia ρ = 0,350. También, entre el nivel de autolesión y cada una de sus dimensiones o componentes: autorregulación ρ = 0,861; búsqueda de sensaciones y fortaleza ρ = 0,767; evitación del suicidio ρ = 0,732; venganza ρ = 0,643; autodominio ρ = 0,700; manifestación de angustia ρ = 0,898 y autolesión y embotamiento ρ = 0,702 (Tabla 3).

**Tabla 3.** **Índice de correlación de las Variables Ansiedad, Depresión y Autolesión con sus dimensiones (a)**.

	Ansiedad (STAID)	Depresión (BDI)	Autolesión (ISAS)	Búsqueda de sensaciones/fortaleza	Autorregulación	Evitación del suicidio	Venganza	Autodominio	Manifestación de Angustia	Embotamiento
Ansiedad (STAID)	1									
Depresión (BDI)	0,467**	1								
Autolesión (ISAS)	0,480**	0,429**	1							
Búsqueda de sensaciones/fortaleza	0,340*	0,138	0,767**	1						
Autorregulación	0,542**	0,541**	0,861**	0,561**	1					
Evitación del suicidio	0,360**	0,508**	0,732**	0,335*	0,725**	1				
Venganza	0,301*	0,175	0,643**	0,510**	0,414**	0,286**	1			
Autodominio	0,309*	0,222	0,700**	0,506**	0,428**	0,389**	0,371**	1		
Manifestación de Angustia	0,350	0,392**	0,898**	0,563**	0,740**	0,660**	0,649**	0,582**	1	
Embotamiento	0,240	0,196	0,702**	0,446**	0,479**	0,395**	0,665**	0,665**	0,649**	1

Nota. Autoría propia a partir de los datos obtenidos en el proceso de investigación. ** La correlación es significativa en el nivel 0,01 (bilateral). * La correlación es significativa en el nivel 0,05 (bilateral).

Se observaron también relaciones positivas y significativas entre el nivel de autolesión y el perfil sensorial evitador de emociones con un ρ = 0,419; entre el perfil sensorial general y la autorregulación ρ = 0,345 y la evitación de suicidio ρ = 0,370; entre la escala de sensibilidad sensorial y la evitación de suicidio ρ = 0,424. Así mismo, entre la sensibilidad sensorial y la autorregulación se observa una correlación positiva de ρ = 0,330. Finalmente, en la relación del evitador de sensaciones y la autorregulación se observó una relación positiva y significativa de ρ = 0,486 y no significativa con el perfil de evitación de suicidio de ρ = 0,561. Sin embargo, con la manifestación y la angustia la relación fue significativa de ρ = 0,343.

## 4. Discusión 

Como ha sido señalado por la organización mundial de la salud (OMS) [21], se ha establecido una relación moderada entre la ansiedad y la depresión. A través de esta investigación, se evidencia que dicha relación no se limita únicamente a personas neurotípicas, sino que también se manifiesta en individuos con autismo, específicamente en adolescentes.

En otras palabras, las asociaciones previamente establecidas entre los trastornos de ansiedad y depresión a nivel global también se observan en la población diagnosticado con TEA. Sin embargo, nuestra investigación ha revelado que no existe una asociación significativa con respecto al género. En consecuencia, se puede afirmar que tanto mujeres como hombres con TEA tienen la misma probabilidad de experimentar ansiedad y/o depresión. Se ha observado, además, que los jóvenes con autismo que experimentan ansiedad pueden experimentar afectaciones en áreas como el autodominio, manifestación de angustia y la evitación del suicidio. En consecuencia, se puede inferir que la ansiedad en adolescentes con TEA está directamente relacionada con la defensividad sensorial, aunque esta relación tiende a ser de baja magnitud. En cuanto a la conducta de autolesión, la investigación ha permitido observar que, a medida que varía esta conducta, también lo hace el perfil sensorial del individuo o adolescente con TEA, específicamente en aspectos como la evitación de emociones, la autorregulación y la evitación del suicidio. En este sentido, y respaldando hallazgos anteriores [13, 22], se evidencia que la autolesión guarda una estrecha relación con la sensibilidad sensorial del individuo, especialmente en lo que respecta a la evitación de emociones y la manifestación de angustia.

En última instancia, se puede concluir que, para el grupo de adolescentes con TEA que participaron en la investigación, no se encontró una correlación positiva entre el grado de ansiedad y/o depresión y el nivel de afectación del autismo. No obstante, sí se identifica una asociación entre los niveles de ansiedad y depresión y el grado de autolesión. Específicamente, se observa que, a mayores niveles de ansiedad o depresión, el individuo con TEA presenta mayores conductas de autolesión. Además, se evidenció una relación positiva entre los grados de autolesión y la defensividad sensorial, específicamente en el perfil de evitador de emociones, indicando que las autolesiones aumentan debido a la intención del individuo con TEA de evitar expresar sus emociones.

## 5. Conclusiones

Los resultados de este estudio evidencian que, existe una asociación significativa entre los adolescentes con TEA y los niveles de ansiedad y depresión, al igual que en la población neurotípica. Sin embargo, no se observaron diferencias en función del género ni una relación directa entre estas variables y el grado de afectación del autismo. En cambio, se hallaron asociaciones sólidas entre la ansiedad, la depresión y las conductas autolesivas, lo que sugiere que, a mayor malestar emocional, mayor es la probabilidad de autolesión en esta población.

Asimismo, se identificó una correlación significativa entre las autolesiones y ciertos perfiles de sensibilidad sensorial, especialmente el evitador de emociones. Esto indica que algunos adolescentes con TEA podrían recurrir a la autolesión como estrategia para gestionar o evitar la expresión emocional y la sobrecarga sensorial. Las dimensiones más destacadas de la autolesión fueron la autorregulación, la manifestación de angustia y la evitación del suicidio, lo que refuerza la idea de que estas conductas cumplen una función psicológica compleja y no necesariamente suicida.

Estos hallazgos subrayan la importancia de evaluar de manera integral el perfil emocional y sensorial en adolescentes con TEA, especialmente en contextos de hospitalización, con el fin de diseñar estrategias de intervención más ajustadas a sus necesidades. No obstante, es recomendable seguir investigando con muestras amplias y herramientas adaptadas para la población con TEA, a fin de comprender mejor los mecanismos subyacentes a la autolesión y ofrecer abordajes terapéuticos más eficaces y personalizados.

## Data Availability

Los datos utilizados en este estudio están disponibles previa solicitud razonada al autor correspondiente.

## Contribuciones de los Autores

BMB ha sido la autora principal del estudio y responsable del diseño, desarrollo y redacción del artículo. AMGH, en su rol de directora de tesis, ha supervisado todo el proceso de investigación, proporcionando orientación metodológica, revisión crítica del contenido y apoyo académico continuo. AGT, como director clínico de Clariane e ITA, ha facilitado el acceso a la muestra de pacientes y ha colaborado en la orientación clínica y metodológica del estudio, contribuyendo con su experiencia en el ámbito de la salud mental. Todos los autores contribuyeron a los cambios editoriales del manuscrito. Todos leyeron y aprobaron el manuscrito final. Todos los autores participaron lo suficiente en el trabajo y aceptaron ser responsables de todos sus aspectos.

## Aprobación Ética y Consentimiento Informado

El estudio “Alteraciones conductuales en adolescentes con trastorno del espectro autista en unidad de hospitalización” ha sido aprobado por el Comité de Ética para la Investigación de ITA Salud Mental en la sesión ordinaria celebrada el 4 de enero de 2021. La evaluación se realizó conforme a los principios éticos establecidos en la Declaración de Helsinki (2013) y la Ley 14/2007 de Investigación Biomédica. El comité consideró que el protocolo presentado cumplía con los requisitos de confidencialidad, autonomía, no maleficencia y beneficencia. Todos los sujetos dieron su consentimiento informado para su inclusión antes de participar en el estudio.

## Agradecimientos

Deseamos expresar nuestro más sincero agradecimiento a los pacientes que participaron en este estudio por su colaboración y predisposición. Asimismo, agradecemos profundamente a todos los compañeros (además del autor) de trabajo por compartir sus conocimientos y contribuir al desarrollo de este artículo.

## Financiación

Esta investigación no recibió financiación externa.
